# Whole body magnetic resonance imaging in newly diagnosed multiple myeloma: early changes in lesional signal fat fraction predict disease response

**DOI:** 10.1111/bjh.14401

**Published:** 2016-10-21

**Authors:** Arash Latifoltojar, Margaret Hall‐Craggs, Neil Rabin, Rakesh Popat, Alan Bainbridge, Nikolaos Dikaios, Magdalena Sokolska, Ali Rismani, Shirley D'Sa, Shonit Punwani, Kwee Yong

**Affiliations:** ^1^ Centre for Medical ImagingUniversity College LondonLondonUK; ^2^ Department of Clinical RadiologyUniversity College London HospitalsLondonUK; ^3^ Department of HaematologyUniversity College London HospitalsLondonUK; ^4^ Department of Medical PhysicsUniversity College London HospitalsLondonUK; ^5^ UCL Cancer Institute, HaematologyUniversity College LondonLondonUK

**Keywords:** magnetic resonance imaging, whole body, multiple myeloma, bortezomib, treatment response

## Abstract

Cross‐sectional imaging techniques are being increasingly used for disease evaluation in patients with multiple myeloma. Whole body magnetic resonance imaging (WB‐MRI) scanning is superior to plain radiography in baseline assessment of patients but changes following treatment have not been systematically explored. We carried out paired WB‐MRI scans in 21 newly diagnosed patients prior to, and 8‐weeks after, starting chemotherapy, and analysed stringently selected focal lesions (FLs) for parametric changes. A total of 323 FLs were evaluated, median 20 per patient. At 8 weeks, there was a reduction in estimated tumour volume (eTV), and an increase in signal fat fraction (sFF) and apparent diffusion coefficient (ADC) in the group as a whole (*P* < 0·001). Patients who achieved complete/very good partial response (CR/VGPR) to induction had a significantly greater increase in sFF compared to those achieving ≤ partial response (PR;* P* = 0·001). When analysed on a per‐patient basis, all patients achieving CR/VGPR had a significant sFF increase in their FL's, in contrast to patients achieving ≤PR. sFF changes in patients reaching maximal response within 100 days (fast responders) were greater compared to slow responders (*P* = 0·001). Receiver Operator Characteristic analysis indicated that sFF changes at 8 weeks were the best biomarker (area under the Curve 0·95) for an inferior response (≤PR). We conclude that early lesional sFF changes may provide important information on depth of response, and are worthy of further prospective study.

The continued development of novel and highly effective treatment protocols for multiple myeloma (MM) has produced a need for increasingly sophisticated tools to assess response in this cancer. Biochemical measurements of paraprotein and heavy or light chains are global measures, while bone marrow‐based techniques have the disadvantage of random sampling, and neither modality addresses the spatial heterogeneity of this disease. Radiological techniques have long been a part of disease assessments; plain radiographic skeletal survey and magnetic resonance imaging (MRI) of the spine have an established role in the diagnosis, staging and assessment of insufficiency fracture risk of patients (Durie *et al*, 2006; D'Sa *et al*, 2007; Hanrahan *et al*, 2010). The use of cross‐sectional imaging techniques [computed tomography (CT), positron emission tomography (PET)‐CT and MRI] are confirmed to be more sensitive than plain radiography in the detection of bone lesions (Zamagni *et al*, 2007; Bartel *et al*, 2009; Regelink *et al*, 2013; Waheed *et al*, 2013). Importantly, the detection of more than one focal lesion (FL) on cross‐sectional imaging is now considered to fulfill the criteria for symptomatic disease deserving of treatment (Rajkumar *et al*, 2014). Furthermore, advanced imaging techniques have also been described to have clinical utility in prognostication. For instance, the number of FLs and metabolic response on PET‐CT following treatment were found to have independent prognostic value in newly diagnosed patients (Zamagni *et al*, 2011, 2015; Moreau *et al*, 2014).

There is growing evidence to support the use of whole body MRI (WB‐MRI) in the diagnostic work up of patients with suspected or confirmed diagnosis of MM (Dutoit *et al*, 2013; Dimopoulos *et al*, 2015). For example, WB diffusion weighted (DW)‐MRI is more sensitive than plain radiographs in picking up FLs in patients with relapsed disease (Giles *et al*, 2015). MRI also provides valuable information on the degree and pattern of marrow involvement, which may also carry prognostic information. A diffuse pattern of marrow involvement or high number of FLs on MRI is associated with inferior outcomes in patients with symptomatic MM (Walker *et al*, 2007; Mai *et al*, 2015) whilst the detection of FLs in patients with otherwise asymptomatic disease alters their risk profile (Hillengass *et al*, 2010). Recently published guideline from the National Institute for Health and Care Excellence (NICE) recommends WB‐MRI for the investigation of patients with suspected plasma cell disorders (NICE 2016).

Whilst initial disease evaluation in MM patients is improved through the application of WB‐MRI, current response parameters remain based on global measures: serum and urine M‐protein levels, serum free light chain ratio and bone marrow evaluation for clonal plasma cells, supplemented by minimal residual disease assessment by flow cytometry or molecular techniques. Recently, functional MRI techniques, such as contrast enhanced (CE) (Lin *et al*, 2010), DW imaging (DWI) (Hillengass *et al*, 2011) and fat‐water imaging (Takasu *et al*, 2012), have become widely available on clinical MRI scanners, providing an opportunity to explore these techniques for response assessment. By detecting intra‐ and extracellular water flux, DWI provides a measure of tissue cellularity and changes in apparent diffusion coefficient (ADC) have been associated with treatment response (Hillengass *et al*, 2011; Giles *et al*, 2014). CE imaging is used to highlight abnormal vasculature, allowing detection of alterations of vasculature following therapy (Lin *et al*, 2010). Involvement by MM lesions leads to changes in haematopoietic (red) and fatty (yellow) marrow, that can be quantified by fat‐water MRI techniques (Takasu *et al*, 2015). In patients with symptomatic MM, signal fat fraction (sFF) of lumbar spine was reported to be significantly lower when compared to patients with asymptomatic disease (Takasu *et al*, 2015). Response to treatment may therefore be expected to lead to an increase in the sFF of involved bone, but such changes have not been investigated as a response measure. The aim of this study was to explore and compare the use of DWI, CE and sFF functional MRI measures, in the evaluation of newly diagnosed MM patients prior to, and soon after starting chemotherapy.

## Materials and methods

### Patient cohort and study design

The study was conducted in accordance with the Declaration of Helsinki and was approved by national research ethics committee. Patients were included if they had a suspected diagnosis of symptomatic multiple myeloma but were previously untreated, with chemotherapy or radiotherapy and were able to undergo MRI scanning (i.e. no contraindication to MRI). A prospective single‐arm observational design was used to study the patient cohort. Patients were recruited into the study from multi‐disciplinary team meetings and underwent WB‐MRI before starting treatment. A second WB‐MRI scan was performed after two cycles of induction chemotherapy at a median of 8 weeks.

### Laboratory Investigations and interphase FISH

Disease response was assessed after each cycle of chemotherapy using serum and urine M‐protein measurements, and assigned according to International Myeloma Working Group (IMWG) guidelines (Rajkumar *et al*, 2011). Baseline interphase‐fluorescence *in situ* hybridization (FISH) was performed on CD138‐selected plasma cells from bone marrow samples, using probes for *IGH* translocations t(4;14), t(11;14) and t(14;16), del(17p), del(13) and 1p‐/1q+ (Smith *et al*, 2015).

### WB‐MRI technique

Imaging was performed using a single 3·0 T wide‐bore MR scanner (Ingenia; Phillips Healthcare, Best, the Netherlands). Full body coverage (vertex to feet) was obtained through a multi‐station acquisition of contiguous body regions with the manufacturers' head coil, two anterior surface coils and table‐embedded posterior coils. Whole‐body coronal pre‐contrast mDixon imaging was complimented by axial T2 weighted turbo spin echo (TSE), axial DWI (with 4 *b*‐values: b0, 100, 300, 1000) and finally, coronal CE mDIXON imaging (following 20 ml of intravenous (IV) gadoterate meglumine (Dotarem^®^, Guebert, France). The average total scan time was 67 min. Full scanning parameters are summarized in Table 1.

**Table 1:** MRI sequence parameters

Imaging plane	T2‐TSE	mDixon (pre‐ and post‐contrast^a^)	DWI (b0, 100, 300, 1000)
	Transverse	Coronal	Transverse
TE (ms)	80	1·02/1·8	71
TR (ms)	1228	3·0	6371
FOV (mm*mm)	500*300	502*300	500*306
Voxel size (mm*mm)	1*1	2·1*2·1	4*4·2
Number of slices	40	120	40
Slice thickness (mm)	5	5	5
Acquisition matrix	500*286	144*238	124*72
ETL	91	2	39
Acceleration factor (SENSE)	2	2	2·5
Pixel bandwidth (Hz)	537	1992	3369
Scan time (s)	47	17	152

T2‐TSE, T2‐weighted turbo spin echo; mDixon, modified dixon; DWI, diffusion weighted imaging; TE, time of echo; TR, time of repetition; FOV, field of view; ETL, echo train length; SENSE, sensitivity encoding.

a Contrast agent 20 ml intravenous gadoterate meglumine, Dotarem^®^, Guerbet, Villepint, France.

### WB‐MRI image analysis

The skeleton was divided into 10 anatomical locations (skull, cervical spine, shoulder girdle, humorous, chest wall, thoracic spine, lumbar spine, pelvis, femur and lower leg) and FLs at pre‐ and post‐treatment scans were localized by two radiologists (MHC and SP with more than 20 and 10 years experience in MR imaging, respectively) in consensus, without knowledge of the results of all other imaging and non‐imaging investigations. The pattern of involvement was noted for each patient as previously described using combination of available sequences (Baur‐Melnyk *et al*, 2005).

On DWI, FL's were identified as focal areas of restricted diffusion returning high signal intensity on DWI b1000 images compared to surrounding marrow, whilst on post‐contrast images, FL's were identified as focal areas of increased contrast uptake compared to surrounding marrow. A 5‐point Likert scale was used to score confidence of an identified lesion representing myeloma (0 = non‐diagnostic quality images, 1 = unlikely, 2 = indeterminate, 3 = likely and 4 = highly likely disease). Likert scoring systems are commonly employed in radiological research and clinical practice (Rosenkrantz *et al*, 2013). Scoring was performed on both DWI and post‐contrast scans, and only matched lesions on both images were selected for analysis as explained below.

To provide an objective per‐patient global measure of disease, and for following treatment response, a maximum of 20 FLs per patient were selected for quantitative analysis based on the following sequentially applied rules:

1. Lesions scored as 3 or 4 visible on matched DWI b1000 and post‐contrast mDixon MRI were selected
2. Lesions < 5 mm in diameter were excluded (as their size precludes accurate quantitation)
3. All lesions from locations containing 1 or 2 lesions ≥5 mm were included
4. The largest lesions from those remaining were selected to make up the maximum of 20 FLs per patient

### WB‐MRI quantitative analysis

A region of interest (ROI) analysis for all selected lesions was performed using Osirix (Version 4·0, Apple, Cupertino, CA, USA). Quantitative biomarkers were estimated by single slice analysis of FLs on slice depicting their largest dimension for both scanning time‐points as below:

#### Estimated tumour volume (eTV)

Using the caliper tool, a 3‐axis measurement of each selected FL was performed on coronal post‐contrast mDixon and axial b1000 DWI images. Individual lesion volume was derived by (X*Y*Z/2).

#### Enhancement ratio (ER)

Using the ROI tool, selected lesions were carefully contoured on post‐contrast water only mDixon images and the average signal intensity (SI) for each FL derived (SI_post‐contrast−W_). The ROI was then transferred to pre‐contrast water only mDixon and average SI derived (SI_pre‐contrast−W_). The signal intensities for individual FL from the pre‐ and post‐contrast water only mDixon images were used to calculate ER as follows: 100*(SI_post‐contrast−W_−SI_pre‐contrast−W_)/SI_pre‐contrast−W_.

#### Apparent diffusion coefficient (ADC)

Using the ROI tool, selected FLs were carefully contoured on the b1000 DW image depicting their maximum diameter. The contours were copied and pasted to the corresponding b0, b100 and b300 DW images and average SI derived for each *b*‐value. The ADC of each selected FL was derived using a mono‐exponential curve fit of signal intensity verses *b*‐value, within MATLAB 2011a (MathWork, Natick, MA, USA) (Punwani *et al*, 2010).

#### Signal fat fraction (sFF)

Using the ROI tool, contoured ROIs on pre‐contrast water only mDixon images (SI_pre‐contrast−W_) were copied and pasted to the corresponding pre‐contrast fat only mDIXON images (SI_pre‐contrast−F_). sFF was calculated by SI_pre‐contrast−F_/(SI_pre‐contrast−F_ + SI_pre‐contrast−W_) (Messiou *et al*, 2012).

Finally, as an internal control, sFF of normal appearing femoral greater trochanter was assessed by a 3 cm^3^ circular ROI at each time point.

### Statistical analysis

Statistical analysis was performed using Prism software (Prism Version 6·0, GraphPad Software, Inc., San Diego, CA, USA). The normality of biomarkers distribution was assessed by Kolmogorov–Smirnov test. Baseline eTV, ER, ADC and sFF for each patient was correlated with age, sex, International Staging System (ISS), Beta‐2 microglobulin, genetic risk and response at end of induction therapy. Spearman's rank correlation coefficient (*r)* was calculated and significance defined as *P* < 0·05. For temporal changes of MRI parameters, a two‐tailed Wilcoxon matched‐pairs signed rank test was used for paired samples that were not normally distributed. For normally distributed data, a two‐tailed paired *t*‐test was used for paired samples, and unpaired *t*‐test for independent samples.

Receiver operating characteristics (ROC) curves, for prediction of patients achieving ≤ PR, were derived for percentage change in each MRI biomarker following treatment [(8 week−baseline) × 100/baseline]. The area under the curve (AUC) was quantified to assess performance for predicting patients achieving ≤ PR across all possible diagnostic thresholds.

## Results

### Patient treatment and disease response

Twenty‐six patients were identified with symptomatic multiple myeloma according to IMWG criteria (Durie *et al*, 2006). One patient had no measurable FL on WB‐MRI and was excluded from further analysis. Demographic and disease characteristics of the remaining 25 patients together with details of induction regimens are shown in Table 2. These included PAD (Bortezomib, Doxorubicin, Dexamethasone; 17 patients), VTD (bortezomib, thalidomide, dexamethasone; 4 patients), CVD (cyclophosphamide, bortezomib, dexamethasone; 3 patients) and MPV (melphalan, prednisone, bortezomib; 1 patient). These regimens were administered as either 3‐ or 4‐week cycles. Patients received a total of 4–6 cycles of induction, with response assessment at the start of each cycle and at the end of induction. Thirteen patients achieved a complete response (CR) or very good partial response (VGPR) to their first line of therapy, while the rest achieved partial response (PR, *n* = 5), minimal response (MR, *n* = 4) or stable disease/progressive disease (SD/PD, *n* = 3).

**Table 2:** Patient demographics, disease parameters and treatment

Patient characteristic (*N* = 25)	Number or median (range)
Age, years	55 (36–72)
Sex, male/female:	15/10
Chain isotype	
IgG	16
IgA	5
Light chain	4
ISS stage	
I	10
II	11
III	4
DS‐PLUS stage	
I	1
II	3
III	21
Induction regimen	
PAD	17
CVD	3
VTD	4
MPV	1
Bone marrow percentage plasma cells	65 (13–90)
Beta‐2 microglobulin (mg/l)	3·5 (1·3–11·3)
Albumin (g/l)	40 (30–53)
Creatinine	79 (56–105)
Genetic risk group	
Standard risk	16
High risk	9

ISS, international staging system; DS‐PLUS, durie‐salmon PLUS staging; PAD, bortezomib, doxorubicin, dexamethasone; CVD, cyclophosphamide, bortezomib, dexamethasone; VTD, bortezomib, thalidomide, dexamethasone; MPV, melphalan, prednisone, bortezomib.

### Baseline WB‐MRI evaluation and Lesion selection

Eight patients had a diffuse/focal pattern and 17 patients had focal only pattern of involvement. On DWI, a median of 51 (range 1–139) FLs was identified for each patient, whilst 24 (range 2–128) FL's were identified on post‐contrast scans,. For parametric analysis, a total of 394 FLs, that were matched on DWI and post‐contrast images [median of 20 FLs per patient (range 1–20)] were evaluated for the entire cohort of 25 patients prior to chemotherapy. There was no significant correlation between number of FLs, or any of the baseline MRI parameters, with patient or disease features, including sex, age, ISS stage, Beta‐2 microglobulin and genetic risk. There was a statistically significant negative correlation between baseline sFF and depth of disease response at the end of induction chemotherapy (Table S1). Patients who achieved CR/VGPR had lower sFF in their FLs [median 0·25 arbitrary units (a.u.), interquartile range 0·21–0·38] compared to those who achieved PR or less (0·35 a.u., interquartile range 0·29–0·49) (*r* = 0·59, *P* = 0·002). There was also a weak positive correlation (*r* = 0·40, *P* = 0·04) between number of FLs on post‐contrast MRI and depth of the response achieved after induction (see Table S1).

### Early mid‐treatment scan

Out of twenty‐five patients with measurable FLs on WB‐MRI, 22 patients attended for an early mid‐treatment scan after 2 cycles of treatment, at a median of 8 weeks from the baseline scan. Of the three patients who did not attend for a second scan, one could no longer tolerate the WB‐MRI due to bortezomib‐related neuropathy; one underwent intra‐medullary nailing of femoral neck and could not be re‐imaged at the defined time point; and one withdrew from the study. Of the remaining 22 patients, one patient received interval radiotherapy and was excluded from the analysis. Temporal changes of WB‐MRI parameters were assessed in the remaining 21 patients.

### Changes in quantitative WB‐MRI parameters at 8 weeks

**Figure 1:**
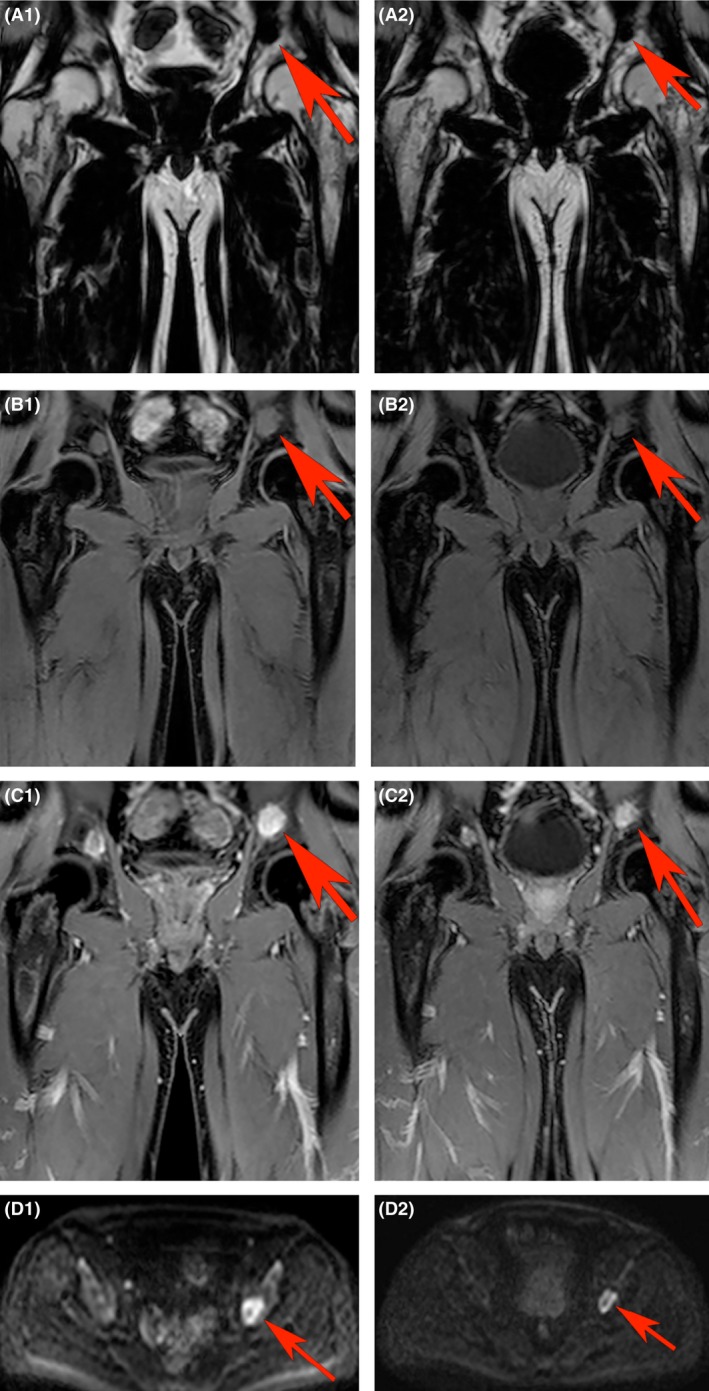
Representative images of a focal lesion (FL) in left pelvis showing changes after 8 weeks. A FL (red arrow) on (A) pre‐contrast fat‐only, (B) pre‐contrast water‐only, (C) post‐contrast water only mDixon and (D) b1000 diffusion weighted imaging at baseline (A1–D1) and 8 weeks (A2–D2) in a patient who achieved very good partial response (VGPR) after induction chemotherapy. Estimated tumour volume (eTV), signal fat fraction (sFF), enhancement ratio (ER) and apparent diffusion coefficient (ADC) were 4·5 cm^3^, 0·11 a.u., 167·8% and 1·13 × 10^−3^ mm^2^/s at baseline, respectively. At 8 weeks after starting chemotherapy, eTV, sFF, ER and ADC were 3·96 cm^3^, 0·23 a.u., 197·5% and 1·97 × 10^−3^ mm^2^/s, representing a 12% reduction in eTV, 109% increase in sFF, 17·6% increase in ER and 74·3% increase in ADC. [Colour figure can be viewed at wileyonlinelibrary.com].

**Figure 2:**
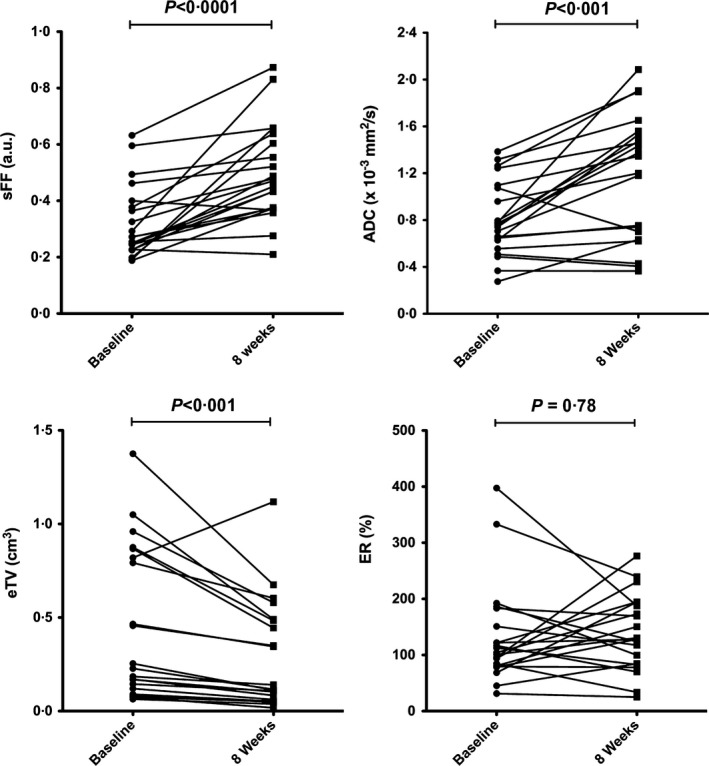
Temporal changes in MRI parameters across entire cohort. Signal fat fraction (sFF), apparent diffusion coefficient (ADC), estimated tumour volume (eTV) and enhancement ratio (ER) at 8 weeks compared with baseline across the entire cohort (*n* = 21). Data for each patient (median for each parameter) are given. Wilcoxon signed‐matched rank test was used to assess temporal changes of MRI parameters and *P* < 0·05 defined as significant.

A total of 323 FLs were evaluable for pre‐ and post‐treatment analysis. Figure 1 shows a pelvic FL at baseline and after 8 weeks of treatment, illustrating change in lesional size and appearance. Summarizing changes across the entire cohort of 21 patients, eTV decreased significantly (from median 0·23 cm^3^ at baseline to 0·12 cm^3^ at 8 weeks, *P* < 0·001), while there was no significant change in ER (median baseline and 8 weeks ER, 104·3% and 128·2%, respectively, *P* = 0·78). Significant increases in both ADC and sFF were seen across the entire cohort; ADC increased from 0·75 × 10^−3^ mm^2^/s to 1·34 × 10^−3^ mm^2^/s (*P* < 0·001) and sFF increased from 0·27 a.u. to 0·47 a.u. (*P* < 0·001) at 8 weeks (Fig 2).

### Correlation of WB‐MRI parameters with IMWG disease response

Of the 21 patients with baseline and 8‐week scans available for analysis, 12 patients achieved CR/VGPR to their first line regimen, while disease responses in the remaining 9 were PR (*n* = 3), MR (*n* = 4), SD (*n* = 1) and PD (*n* = 1). One patient who had biochemically‐defined PR had residual heavy disease burden in the bone marrow (90%) and was re‐classified as MR. Details of these patients are given in Table S2. A total of 208 (median 20 range 4–20) FLs in the CR/VGPR group and 115 (median11 range 1–20) FLs in the ≤PR group were evaluated. The changes in each parameter for these two groups of patients are summarized in Table 3, and Figure S1. Significant changes in eTV, sFF and ADC (*P* < 0·001 for all) were noted for both groups, but no significant change was observed in ER for either group.

**Table 3:** Baseline and 8 week imaging parameters (median and interquartile range, IQR) for CR/VGPR and ≤PR patients. Temporal changes in each group were assessed using Wilcoxon matched‐pairs signed rank test and significance defined as *P* < 0·05

	Scan	Median eTV (IQR)	Median ER (IQR)	Median ADC (IQR)	Median sFF (IQR)
CR/VGPR (*n* = 12)	Baseline	0·24 (0·15–0·86)	114·7 (97·2–190·2)	0·79 (0·66–1·26)	0·24 (0·20–0·27)
	8 weeks	0·13 (0·10–0·47)^a^	140·6 (88·4–184·2)	1·50 (0·88–1·83)^a^	0·44 (0·37–0·63)^a^
≤ PR (*n* = 9)	Baseline	0·17 (0·08–0·87)	84·8 (73·6–121·9)	0·66 (0·52–0·87)	0·40 (0·29–0·54)
	8 weeks	0·08 (0·04–0·59)^a^	127·8 (56·0–194·5)	0·75 (0·51–1·27)^a^	0·48 (0·32–0·60)^a^

CR, complete response; VGPR, very good partial response; PR, partial response; eTV, estimated total tumour volume; ER, enhancement ratio; ADC, mean apparent diffusion coefficient; sFF, signal fat fraction; IQR, interquartile range.

a Significant change (*P* < 0·05) compared with baseline scan.

**Figure 3:**
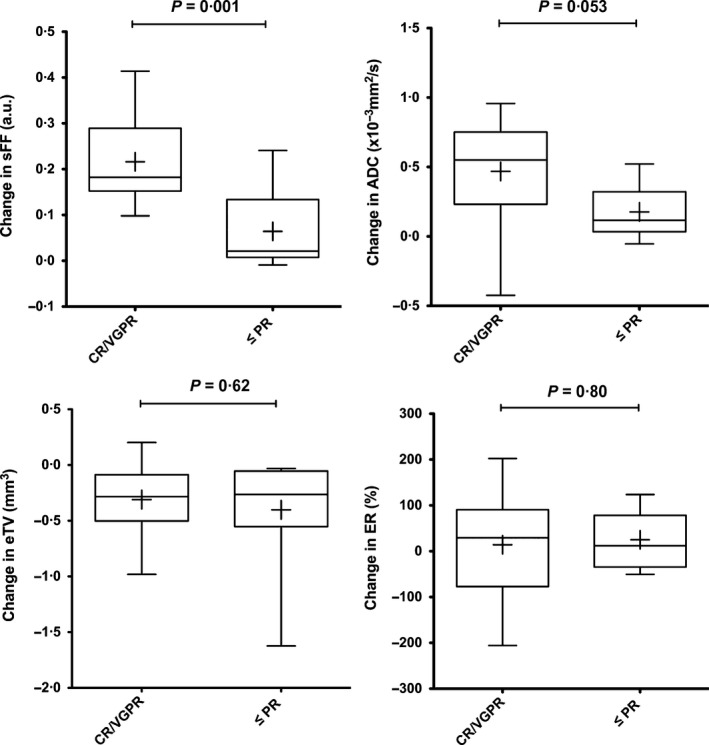
Comparison of changes in MRI parameters between response groups. Changes in signal fat fraction (sFF), apparent diffusion coefficient (ADC), estimated tumour volume (eTV) and enhancement ratio (ER) were compared between the CR/VGPR and ≤PR patient groups. Box and whisker plot of changes in each parameter [(8 weeks)−(baseline)] for each group. The boundaries of the box show 25th and 75th percentiles, and the line within the box is the median. Whiskers show 10th and 90th percentiles. Means are shown (+).An unpaired *t*‐test was used to compare changes between the two patient groups and *P* < 0·05 was defined as significant. CR, complete response; VGPR, very good partial response; PR, partial response.

We then compared the magnitude of change in eTV, sFF between the CR/VGPR and the ≤PR patient groups. While there was a significant reduction in eTV in both groups following treatment, the magnitude of change was similar (*P* = 0·62). On the other hand, the magnitude of change in sFF was significantly greater in the CR/VGPR (+0·21 ± 0·06, mean ± SD) compared with the ≤PR group (+0·06 ± 0·08, *P* = 0·001). Although the changes in ADC were greater in the CR/VGPR group (+0·47 ± 0·39) compared with the ≤PR group (+0·18 ± 0·18), this did not reach significance (*P* = 0·053) (Fig 3).

As internal controls for each patient, we also evaluated sFF of uninvolved greater trochanteric ROI. Two patients from the ≤PR group were excluded from this analysis due to diffuse involvement of greater trochanter and artefact on MR images. There was no significant change in greater trochanteric sFF in the CR/VGPR group (*n* = 12), and the ≤PR group (*n* = 7) (Figure S2).

### Consistency and speed of lesional responses

**Figure 4:**
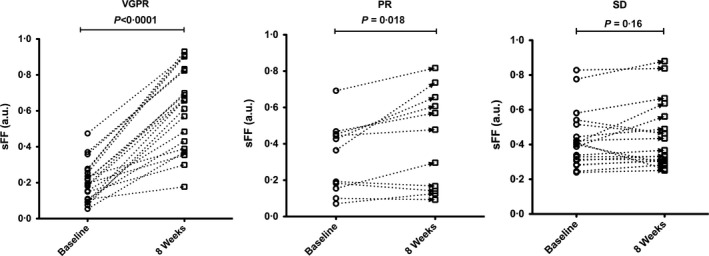
Lesional signal fat fraction (sFF) changes for representative patients achieving very good partial response (VGPR), partial response (PR) and stable disease (SD) following induction chemotherapy. Data points in each graph represent focal lesions at baseline and 8 weeks after starting treatment. To assess the temporal changes, a Wilcoxon matched‐pairs signed rank test was used and *P* < 0·05 was defined as significant.

Data for the sFF of each lesion in representative patients achieving VGPR, PR and SD are shown in Fig 4, where it can be seen that, while all lesions manifest an increase in sFF in the patient with VGPR, responses are more mixed and individual lesional changes more variable in patients who did not achieve a deep biochemical response. Analysing sFF changes in each patient, every patient achieving CR/VGPR had a significant increase in lesional sFF, except for one patient who had only 4 FL's for analysis. One patient with a single FL who achieved PR was excluded from per patient analysis. In contrast to the CR/VGPR group, none of the 6 patients with <PR had a significant change in the sFF of their FLs, whilst two out of the three patients with PR demonstrated a significant increase in sFF. Eight out of the 12 patients achieving CR/VGPR had a significant rise in ADC of their FL's, while 2 out of 6 patients with <PR had a significant rise in ADC of their FL's (Table S3).

**Figure 5:**
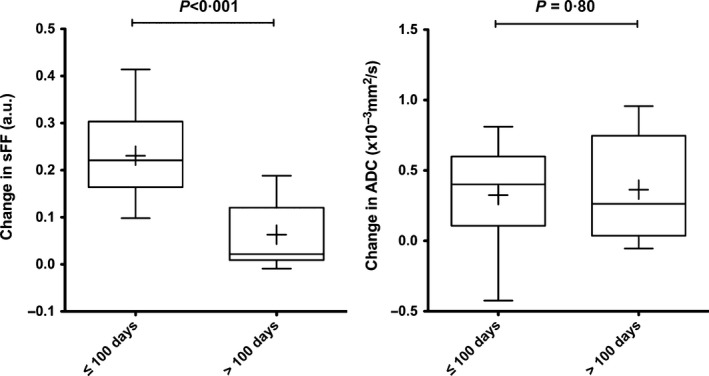
Magnitude of changes in signal fat fraction (sFF) and apparent diffusion coefficient (ADC) according to speed of biochemical response. Changes from baseline [(8 weeks)−(baseline)] were assessed separately for patients who reached their maximal response (lowest paraprotein level) within 100 days (*n* = 11) versus those who took longer than 100 days (*n* = 10). Box and whisker plots of changes in sFF and ADC. The boundaries of the box show 25th and 75th percentiles, and the line within the box is the median. Whiskers show 10th and 90th percentiles. Means are shown (+). An unpaired *t*‐test was used to assess changes in MRI parameters [(8‐week values)−(baseline values)] between the two patient groups and *P* < 0·05 was defined as significant.

We also investigated if WB‐MRI changes at 8 weeks correlated with speed of response. Patients were divided into two subgroups based on the time to maximum response (≤ or >100 days). Patients who reached maximum response ≤100 days (*n* = 11) had significantly greater changes in sFF (8 weeks‐baseline) when compared to those with whose time to maximum response was >100 days (*n* = 10, *P* < 0·001). No significant difference in ADC changes was observed between these two groups of patients (Fig 5).

### Predictive value of sFF and ADC changes

Finally, we carried out ROC analysis to evaluate the predictive value of percentage changes of MRI biomarkers for response quality (CR/VGPR versus ≤ PR). Area under the curve analysis showed an AUC of 0·95 (95% confidence interval 0·87–1·00) for percentage changes of sFF as predictor of ≤ PR whist other MRI biomarkers were less reliable for predicting ≤PR (Figure S3 and Table S4).

## Discussion

In this study, we investigated the possible utility of parametric analysis of WB‐MRI scanning in newly diagnosed MM patients for the assessment of baseline characteristics, as well as for early assessment of treatment response. We observed that patients who achieved major responses (CR/VGPR) had a lower sFF in their FLs compared to patients who achieved ≤ PR. We also observed a significant decrease in eTV at 8 weeks that appeared to be independent of response depth. On the other hand, changes in lesional sFF were significantly greater in patients achieving CR/VGPR, when compared with those whose disease response was PR or less. A trend to similar differences in ADC changes was also observed. The magnitude of change in lesional sFF also seemed to differentiate between fast and slow responders. ROC analysis indicated the superior value of sFF changes at 8 weeks in predicting a major response.

Treatment‐related increases in ADC have previously been reported in MM patients (Messiou *et al*, 2012; Bonaffini *et al*, 2015). Messiou *et al* (2012) assessed ADC of up to five ROIs on ADC maps (including diffuse marrow involvement and normal appearing bone marrow, as opposed to FL only), and observed an initial increase of ADC at 4–6 weeks of treatment in responding patients, followed by a decrease at 20 weeks. These authors also reported that significantly lower percentage of pixels representing fatty marrow in baseline ADC histograms in patients with active myeloma (*n* = 8, 27·2%) compared to patients in remission (*n* = 12, 83·0%). By implementing a two *b* value (b50, 900 s/mm^2^) WB‐DWI on 1·5 T scanner and segmenting all area of visible marrow, Giles *et al* (2014) showed that the ADC increased in 95% (19/20) of responding patients, but that it decreased in all non‐responders (5/5). Given that myeloma is a patchy disease, we selected stringently defined areas of involved skeleton, hence the emphasis on FLs. Horger *et al* (2011) analysed both intra and extramedullary FLs >20 mm and demonstrated a 63·9% increase (range 8·7–211·2%) in their responding cohort compared to 7·8% reduction in ADC in their only non‐responding patient. In line with a previous report, we did not observe any significant change in the enhancement of FLs following treatment (Lin *et al*, 2010). It is possible that changes in angiogenesis within FL are not optimally captured at this early time point of treatment. Persistent increased angiogenesis has been previously reported in MM patients following successful treatment (Rajkumar *et al*, 1999).

Previous reports investigating sFF quantification in bone marrow are scarce. Using iterative decomposition of water and fat with echo asymmetric and least‐squares estimation (IDEAL) Dixon‐based MRI, Takasu *et al* (2015) demonstrated a significant decrease in sFF in the lumbar spine in symptomatic myeloma patients, when compared with asymptomatic multiple myeloma. We have chosen to concentrate our analysis on FL because the presence of FL is recognized to be more relevant (compared to diffuse marrow signal abnormality) to disease pathogenesis and risk assignment (Rajkumar *et al*, 2014), hence signal changes in these diseased areas are likely to be more prognostically relevant. To the best of our knowledge, our work is the first to systematically examine changes in the sFF of FLs following initiation of treatment in newly diagnosed MM patients, to report significant increases in sFF after 8 weeks of treatment and the potential utility of early sFF changes as a predictor of depth of response to induction chemotherapy.

Depth of response in newly diagnosed patients undergoing novel agent‐containing regimens has been shown to correlate with disease‐free and, in some cases, overall survival (Martinez‐Lopez *et al*, 2011; Vij *et al*, 2015). Longer follow‐up of our patient cohort is needed in order to explore an association of MRI response parameters with survival, as has been reported for PET‐CT negativity (Zamagni *et al*, 2011, 2015). Our results also need confirmation in a larger cohort of patients treated prospectively, ideally in a uniform manner. We chose major response (CR/VGPR) category for correlative analysis, because in this era of novel regimens where the majority of patients are likely to achieve at least a PR, the attainment of a deep response is not only achievable in a significant number of patients, but serves as better indicator of long term benefit (Lional & Andeson, 2014). If the superior predictive value of sFF is confirmed in a larger study, the use of WB‐MRI to assess sFF changes early during treatment will aid real time response assessment. By providing an early indicator of response quality, such analyses can be incorporated into risk‐stratified protocols. Such radiological parameters need integration with current platforms for risk assignment that are a composite of baseline features and response depth/quality.

A major attraction of WB‐MRI quantitative parameters is the applicability to patients with non‐secretory or oligo‐secretory disease, who have traditionally not been studied in clinical trials and for whom accurate disease response assessment can be challenging. For such patients, WB‐MRI represents a non‐invasive alternative to repeated bone marrow sampling, with the added advantage of providing more comprehensive information about non‐axial and extra‐medullary disease. We noted that some patients showed lesional variation in the sFF and ADC changes at 8 weeks (see representative patients with PR and SD in Fig 4). While targeted biopsy of individual lesions is required to confirm active disease, the ability to track individual lesions within a patient may provide prognostic information. Evidence is accumulating that MM is a multi‐clonal disease from diagnosis (Bolli *et al*, 2014; Lohr *et al*, 2014; Walker *et al*, 2014), and spatial heterogeneity in radiological response to therapy may reflect sub‐clonal differences in treatment response. The ability to track individual FLs for longitudinal monitoring may be especially valuable in patients harbouring adverse genetic lesions. For such patients, further follow‐up scans to monitor “resistant” lesions may be necessary to pre‐empt rapid disease reactivation.

This study was performed on a single MRI scanner, however, as the MRI pulse sequence used to perform these measurements is routinely available on all scanners, and the quantification steps involved are simple, there should be few barriers to generalization. Despite promising results concerning the application of ADC as MRI biomarker for monitoring response in various soft tissue and skeletal tumours (Punwani *et al*, 2010; Giles *et al*, 2014), generalization of ADC remains challenging (Koh *et al*, 2011). ADC quantitation could be affected by several factors, such as number and choices of *b* values, mathematical model for data fitting, software for data analysis and choices of applied sequence parameters (Koh *et al*, 2011; Celik, 2016). Furthermore, in the bone marrow microenvironment, the temporal changes of ADC following myelomatous infiltration and treatment are complex and affected by several factors, such as cell size, bulk flow in capillaries, cellular architecture and the amount of fatty (yellow) marrow (Messiou & Kaiser, 2015). At the very least, lesional sFF will provide complementary information to ADC, but may well prove superior in reproducibility and practical application. For example, a 10‐min protocol could be performed for sFF quantification of FL to provide a response marker of treatment.

In conclusion, we have demonstrated that the assessment of newly diagnosed patients using WB‐MRI to derive sFF and ADC of stringently selected FLs has potential utility in disease response assessment that will complement currently available techniques. sFF changes appear to be most predictive of deep and rapid responses in newly diagnosed patients undergoing induction therapy. Provided these imaging biomarkers are prospectively validated in larger cohorts, they may prove a valuable addition to the current risk platforms in MM patients, integrating baseline features and treatment response.

## Funding

AL was supported by a CRUK/EPSRC award (C1519/A10331) from the KCL/UCL Comprehensive Cancer Imaging Centre. ND was supported by UK EPSRC grants EP/I018700/1 and EP/H046410/1.

## Authorship

Arash Latifoltojar, Margaret Hall‐Craggs, Shonit Punwani, Kwee Yong: Designed and performed the research study, analysed the data, wrote the paper. Neil Rabin, Rakesh Popat, Ali Rismani, Shirley D'Sa: Performed the research study, wrote the paper. Alan Bainbridge, Magdalena Sokolska: Acquired data, wrote the paper. Nikolaos Dikaios: Analysed the data, wrote the paper.

## Conflicts of Interest

The authors of this manuscript have no conflicts of interest to disclose with regard to the work submitted.

## Supporting information


**Table SI.** Correlation between baseline MRI parameters and depth of response to first line therapy.
**Table SII.** Details of patients with paired scans.
**Table SIII.** Changes in median FF and ADC in individual patients who underwent paired scans.
**Table SIV.** Prognostic performance of signal fat fraction (sFF) changes in CR/VGPR and ≤PR groups.
**Fig S1.** Changes in signal fat fraction (sFF), apparent diffusion coefficient (ADC), estimated tumour volume (eTV) and enhancement ratio (ER) according to response.
**Fig S2.** Signal fat fraction (sFF) changes of femoral greater trochanter in CR/VGPR and ≤PR groups.
**Fig S3.** Receiver operating characteristic curves (ROC) for percentage changes (Δ) in signal fat fraction (sFF), apparent diffusion coefficient (ADC), estimated tumour volume (eTV) and enhancement ratio (ER) as predictor of ≤PR.
