# Supplementary material for: Whole body magnetic resonance imaging in newly diagnosed multiple myeloma: early changes in lesional signal fat fraction predict disease response
Source: Br J Haematol. 2016 Oct 21;176(2):222–33. doi: 10.1111/bjh.14401 (PMC5244686; doi:10.1111/bjh.14401)
Supplement: Supplementary file 1 — Table SI. Correlation between baseline MRI parameters and depth of response to first line therapy. Table SII. Details of patients with paired scans. Table SIII. Changes in median FF and ADC in individual patients who underwent paired scans. Table SIV. Prognostic performance of signal fat fraction (sFF) changes in CR/VGPR and ≤PR groups. Fig S1. Changes in signal fat fraction (sFF), apparent diffusion coefficient (ADC), estimated tumour volume (eTV) and enhancement ratio (ER) according to response. Fig S2. Signal fat fraction (sFF) changes of femoral greater trochanter in CR/VGPR and ≤PR groups. Fig S3. Receiver operating characteristic curves (ROC) for percentage changes (Δ) in signal fat fraction (sFF), apparent diffusion coefficient (ADC), estimated tumour volume (eTV) and enhancement ratio (ER) as predictor of ≤PR. [file BJH-176-222-s001.pdf]

Supplementary Table 1. Correlation between baseline MRI parameters and depth of response to first line therapy.

	Median eTV (IQR)	Median sFF (IQR)	Median ER (IQR)	Median ADC (IQR)	Median DWI FL count (IQR)	Median Post-contrast FL load (IQR)
CR/VGPR (n=13)	0.25 (0.15-1.05)	0.25 (0.21-0.38)	116.6 (98.6-187.9)	0.78 (0.58-1.25)	61 (33.5-84.5)	40 (22-53.5)
≤ PR (n=12)	0.16 (0.08-0.71)	0.35 (0.29-0.49)	89.4 (71.1-124.3)	0.68 (0.57-0.76)	32.5 (25.7-59.7)	22 (13.5-24)
P value summary	0.576	0.002 †	0.335	0.053	0.079	0.046 †
r value summary	0.117	-0.592	0.201	0.391	0.357	0.402

IQR: Interquartile range, **CR**: Complete response, **VGPR**: Very good partial response, **PR**: partial response, **eTV**: estimated tumour volume, **sFF**: Signal fat fraction, **ADC**: Apparent diffusion coefficient, **FL**: focal lesion, **DWI**: Diffusion weighted imaging
† Significant correlation
r: Spearman correlation coefficient

Supplementary Table 2. Details of patients with paired scans.

	CR/VGPR	PR or less
**Patient Characteristic (N=21)**	**Number or Median (range)**	**Number or Median (range)**
**Age**	**58.8 (46-69)**	**51.5 (36-61)**
**Sex**	**Male/Female: 7/5**	**Male/Female: 6/3**
**Chain Isotype**		
IgG	**7**	**7**
IgA	**4**	**0**
Light chain	**1**	**2**
**ISS stage**		
I	**3**	**3**
II	**7**	**5**
III	**2**	**1**
**DS-PLUS Stage**		
I	**0**	**1**
II	**1**	**1**
III	**11**	**7**
**Induction regimen**		
PAD	**9**	**7**
CVD	**1**	**2**
VTD	**2**	**0**
**Bone Marrow plasmacytosis (%)**	**54.2(13-90)**	**59.4 (30-80)**
**Beta-2 Microglobulin (mg/L)**	**4.4 (1.3-10.3)**	**3.3 (1.8-6.4)**
**Creatinine (umol/L)**	**80.2 (58-100)**	**80.3 (58-105)**

ISS: International staging system **DS-PLUS**: Durie-Salmon PLUS **PAD**: Bortezomib, Doxorubicin, Dexamethasone **CVD**: Cyclophosphamide, Bortezomib, Dexamethasone **VTD**: Bortezomib, Thalidomide, Dexamethasone

Supplementary Table 3: Changes in median FF and ADC in individual patients who underwent paired scans. To assess temporal changes of MRI parameters in each patient, a Wilcoxon matched-pairs signed rank test was used and p<0.05 defined as significant changes compared to baseline.

Pt ID	Response	FL no	sFF pre	sFF post	p-value	ADC pre	ADC post	p-value
**ID 1**	CR	20	0.244	0.375	<0.01 †	1.318	1.654	0.03 †
**ID 2**	CR	20	0.188	0.373	<0.01 †	1.244	1.457	0.61
**ID 3**	CR	4	0.272	0.357	0.12	1.385	1.895	0.62
**ID 4**	VGPR	20	0.192	0.658	<0.01 †	0.795	1.473	<0.01 †
**ID 5**	VGPR	8	0.292	0.831	0.02 †	0.275	0.634	0.14
**ID 6**	VGPR	18	0.271	0.373	0.01 †	0.779	2.085	<0.01 †
**ID 7**	VGPR	20	0.226	0.433	<0.01 †	0.756	1.434	<0.01 †
**ID 8**	VGPR	20	0.22	0.488	<0.01 †	1.263	1.905	<0.01 †
**ID 9**	VGPR	20	0.376	0.637	<0.01 †	0.507	0.429	0.69
**ID 10**	VGPR	20	0.198	0.604	<0.01 †	0.628	1.530	<0.01 †
**ID 11**	VGPR	20	0.246	0.432	<0.01 †	0.741	1.560	<0.01 †
**ID 12**	VGPR	18	0.249	0.450	<0.01 †	1.074	0.702	0.01 †
**ID 13**	PR	1	0.632	0.873	N/A	0.647	0.754	N/A
**ID 14**	PR	11	0.364	0.478	0.01 †	0.959	1.265	0.24
**ID 15**	PR	20	0.325	0.470	<0.01 †	1.098	1.345	0.02 †
**ID 16**	MR	11	0.227	0.210	0.63	0.772	1.374	0.03 †
**ID 17**	MR	20	0.255	0.275	0.10	0.706	1.180	0.02 †
**ID 18**	MR	8	0.493	0.554	0.25	0.659	0.741	0.84
**ID 19**	MR	16	0.409	0.589	0.14	0.367	0.365	0.69
**ID 20**	SD	19	0.400	0.367	0.16	0.487	0.405	0.10
**ID 21**	Prog	9	0.595	0.683	0.82	0.55	0.62	0.49

FL no: Focal lesion number assessed, **sFF**: Signal fat fraction, **ADC**: Apparent diffusion coefficient
CR: Complete response, **VGPR**: Very good partial response, **PR**: Partial response
MR: Minimal response, **SD**: Stable disease, **Prog**: Progressive disease
†: Significant change (p<0.05) compared with baseline scan.

Supplementary Table 4: Prognostic performance of signal fat fraction (sFF) changes in CR/VGPR and ≤ PR groups. Percentage changes in sFF [(8 weeks-baseline) * 100] and the percentage (n/total) of patients that fall into 5 different categories are summarized for each group.

Changes in sFF	CR/VGPR (n/total)		≤ PR (n/total)
0-9%	1/12 (8.3%)		6/9 (66.6%)
≥ 10%	11/12 (91.6%)		3/9 (33.3%)
≥ 20%	5/12 (41.6%)		1/9 (11.1%)
≥ 30%	3/12 (25%)		0/9 (0%)
≥ 40%	1/12 (8.3%)		0/9 (0%)

sFF: Signal fat fraction
CR: Complete response
VGPR: Very good partial response
PR: Partial response

Supplementary Figure 1

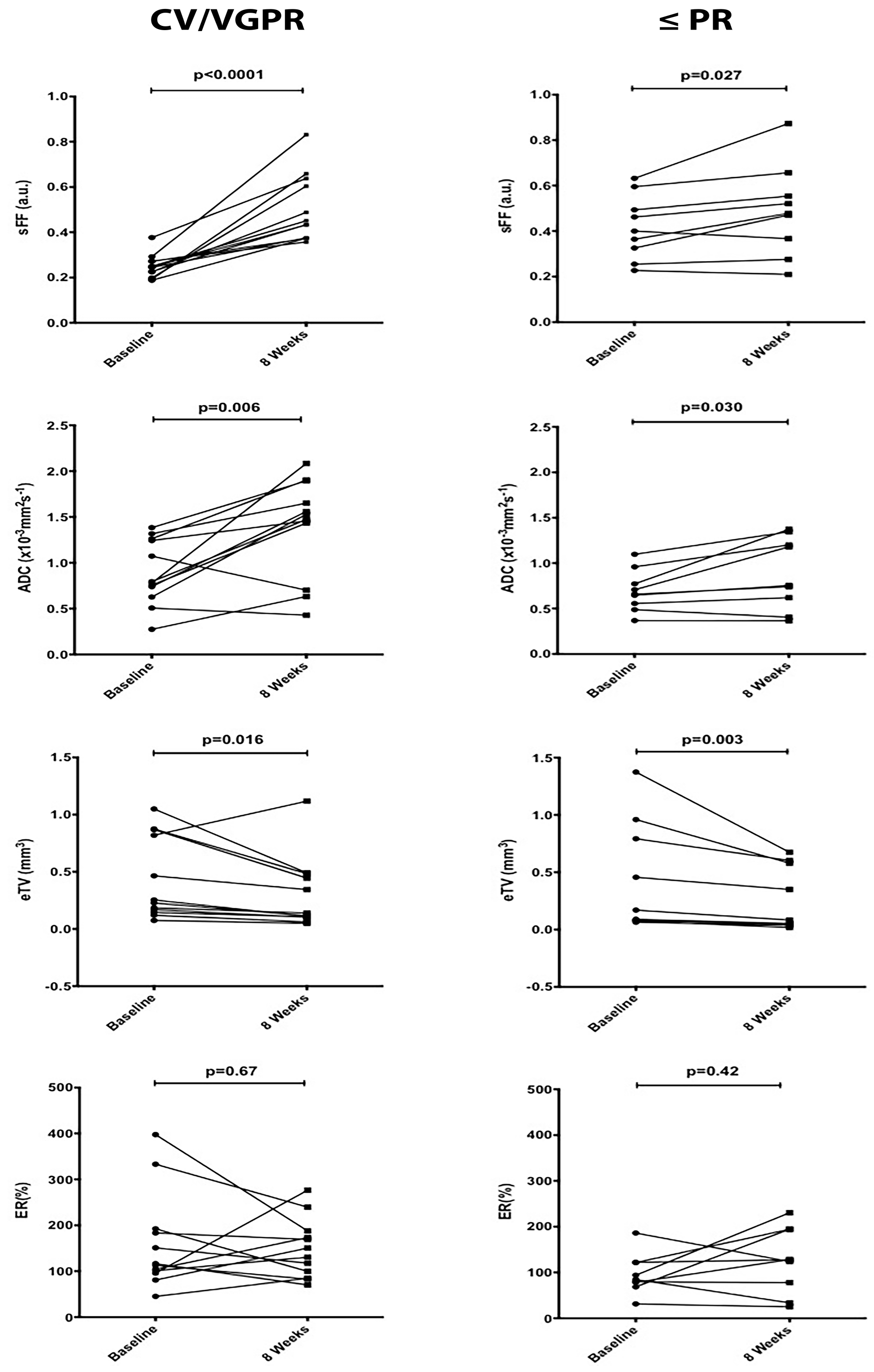

Supplementary Figure 1. Changes in signal fat fraction (sFF), apparent diffusion coefficient (ADC), estimated tumour volume (eTV) and enhancement ratio (ER) according to response. Data (median for each parameter) are given for each patient in the two groups achieving CR/VGPR (n=12) and ≤ PR (n=9).

Wilcoxon signed-matched rank test was used to assess temporal changes of each MRI parameter in each group and $p<0.05$ defined as significant.

Supplementary Figure 2

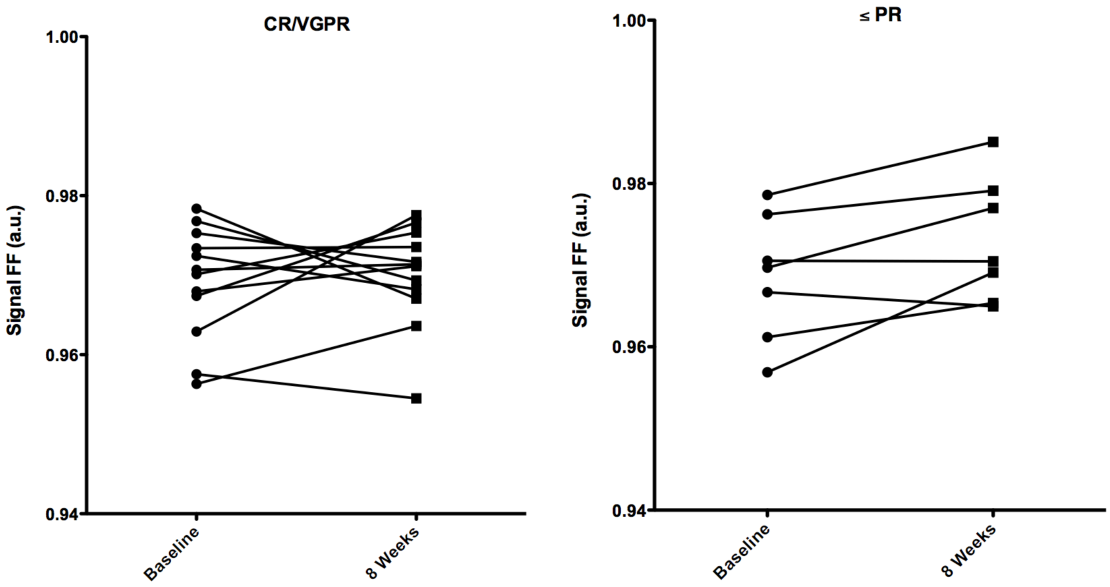

Supplementary Figure 2. Signal fat fraction (sFF) changes of femoral greater trochanter in CR/VGPR and ≤ PR groups. For CR/VGPR group, mean (SD) of sFF were 0.97 (±0.007) and 0.97 (±0.006) at baseline and 8 weeks, respectively (p= 0.73).

For ≤ PR group mean (SD) of sFF were 0.97 (±0.008) and 0.97 (±0.007) at baseline and 8 weeks, respectively (p= 0.078).

Each data point represents sFF measurement of greater trochanter in each patient.

A paired *t*-test was used to assess the temporal changes of femoral greater trochanter's sFF and p<0.05 defined as significant.

Supplementary Figure 3

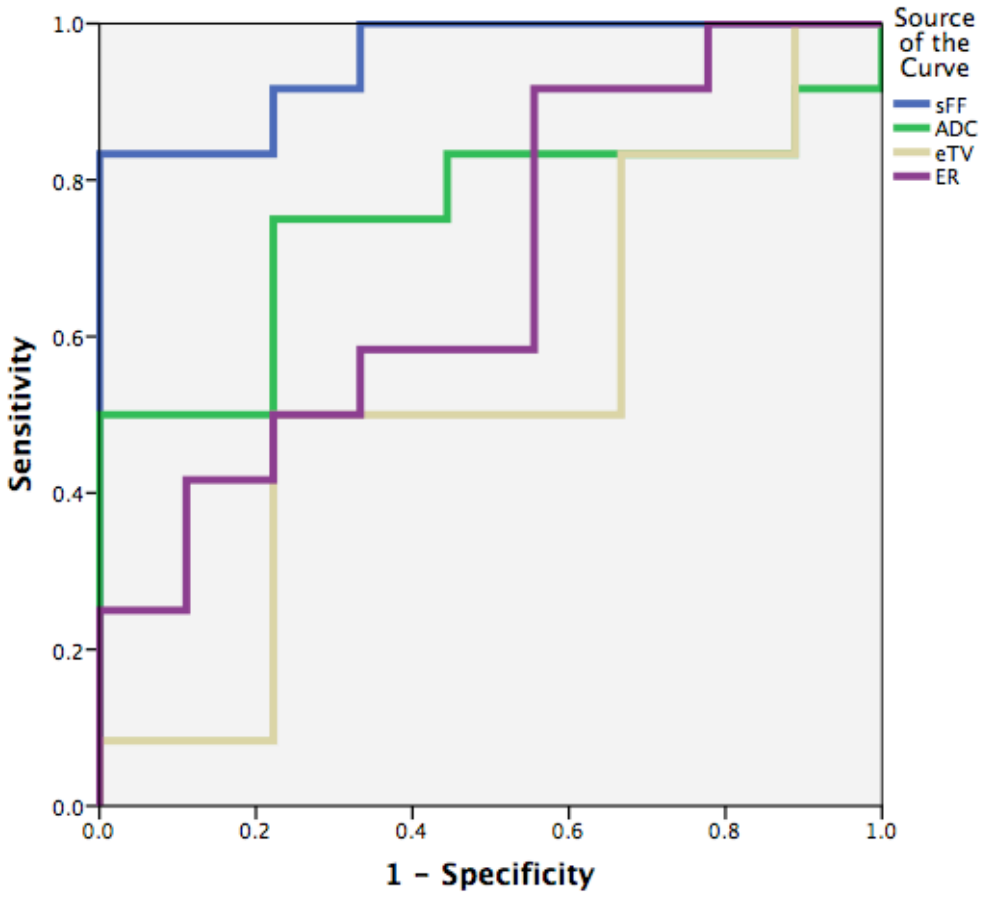

Supplementary Figure 3. Receiver operating characteristic curves (ROC) for percentage changes (Δ) in signal fat fraction (sFF), apparent diffusion coefficient (ADC), estimated tumour volume (eTV) and enhancement ratio (ER) as predictor of ≤ PR. Area under the curve (AUC) and 95% confidence interval (95% CI) are 0.95 (95% CI 0.87-1.00), 0.75 (95% CI 0.53-0.97), 0.54 (95% CI 0.27-0.80) and 0.68 (95% CI 0.45-0.92) for sFF, ADC, eTV and ER, respectively.
